# The contribution of pUL74 to growth of human cytomegalovirus is masked in the presence of RL13 and UL128 expression

**DOI:** 10.1099/jgv.0.000475

**Published:** 2016-08

**Authors:** Kerstin Laib Sampaio, Cora Stegmann, Ilija Brizic, Barbara Adler, Richard J. Stanton, Christian Sinzger

**Affiliations:** ^1^​ Institute of Virology, University of Ulm, Ulm, Germany; ^2^​ Max von Pettenkofer-Institute for Virology, Ludwig-Maximilians-University Munich, Munich, Germany; ^3^​ School of Medicine, University of Rijeka, Rijeka, Croatia; ^4^​ Institute of Infection and Immunity, School of Medicine, Cardiff University, Cardiff, UK

**Keywords:** Cytomegalovirus, glycoproteins, cell-to-cell-spread

## Abstract

The glycoproteins gH and gL of human cytomegalovirus (HCMV) form a complex either with pUL74 (trimeric complex) or with proteins of the UL128 locus (pentameric complex). While the pentameric complex is dispensable for viral growth in fibroblasts, deletion of pUL74 causes a small plaque phenotype in HCMV lab strains, accompanied by greatly reduced cell-free infectivity. As HCMV isolates, shortly after cultivation from clinical specimens, do not release cell-free infectious viruses, we wondered whether deletion of pUL74 would also affect virus growth in this background. To address this question, we took advantage of the bacterial artificial chromosome (BAC)-cloned virus Merlin-RL13_tetO,_ which grows cell associated due to the inducible expression of the viral RL13 gene, thereby resembling clinical isolates. Stop codons were introduced by seamless mutagenesis into UL74 and/or the UL128 locus to prevent expression of the trimeric or pentameric complex, respectively. Virus mutants were reconstituted by transfection of the respective genomes into cultured cells and analysed with respect to focal growth. When the UL128 locus was intact, deletion of pUL74 did not notably affect focal growth of Merlin, irrespective of RL13 expression. In the absence of UL128 expression, foci were increased compared with wild-type, and infectious cell-free virus was produced. Under these conditions, disruption of UL74 completely prevented virus spread from initially transfected cells to surrounding cells. In conclusion the contribution of pUL74 is masked when the UL128 locus is expressed at high levels, and its role in cell-free virus spread is only revealed when expression of the pentameric complex is inhibited.

## Introduction

All herpesviruses depend on complexes of glycoproteins H and L for successful replication, but they vary with respect to the composition of these complexes (Connolly *et al.*, 2011; Eisenberg *et al.*, 2012). While many herpesviruses only form gH/gL heterodimers, betaherpesviruses encode a third complex member, glycoprotein O, resulting in the formation of a gH/gL/gO heterotrimer. In human cytomegalovirus (HCMV) gO is encoded by the UL74 reading frame (Huber & Compton, 1997, 1998; Li *et al.*, 1997), and disruption of the respective protein pUL74 is associated with a reduction in cell-free infectivity and plaque size (Dunn *et al.*, 2003; Hobom *et al.*, 2000; Jiang *et al.*, 2008; Wille *et al.*, 2010; Yu *et al.*, 2003). In addition, gH and gL can form a pentameric complex with the three proteins encoded by the UL128 gene locus, and disruption of this complex restricts the cell tropism of the virus, greatly reducing infection efficiency of epithelial cells, endothelial cells and leukocytes (Adler *et al.*, 2006; Hahn *et al.*, 2004; Ryckman *et al.*, 2008b; Wang & Shenk 2005).

Supported by interference studies, it was assumed that the trimeric and the pentameric gH/gL complex serve the same function on different cell types, e.g. triggering the proprietary fusion protein gB upon binding to the respective receptor (Ryckman *et al.*, 2008a; Vanarsdall *et al.*, 2011, 2012). However, a recent comparison of cell-free virions with different levels of gH/gL complexes in their envelopes strongly suggests that the trimeric complex mediates efficient entry of cell-free virus irrespective of the cell type by promoting the fusion step, whereas the pentameric complex might serve a distinct additional function required only on certain cell types such as endothelial or epithelial cells (Zhou *et al.*, 2015). This is concordant with the notion that deletion of gO reduces cell-free infectivity not only on fibroblasts but also on endothelial and epithelial cells (Jiang *et al.*, 2008; Wille *et al.*, 2010).

While the contribution of gH/gL complexes during the entry of cell-free HCMV is well documented, little data are available regarding their role during cell-associated transmission. To date, accessory proteins have only been knocked out in the genetic background of strains that release cell-free infectious progeny. Under these conditions it is hard to distinguish between effects on cell-free transmission and effects on cell-associated spread.

A differentiation between the two transmission modes matters as clinical isolates grow cell-associated in cell culture after primary isolation from patient material (Sinzger *et al.*, 1999), and cell-to-cell spread is, hence, regarded as a relevant means of HCMV dissemination *in vivo*. With continuous passaging of HCMV isolates, disrupting mutations in RL13 and the UL128 locus emerge, leading to release of cell-free infectivity (Dargan *et al.*, 2010). These alterations occur within a few passages and obviously provide a strong growth advantage as it is impossible to reconstitute and maintain a genetically complete virus in cell culture unless both gene regions are protected against this selective pressure by conditional repression (Dargan *et al.*, 2010; Stanton *et al.*, 2010).

We used a genetically complete HCMV, generated using the bacterial artificial chromosome (BAC)-cloned strain Merlin, to revisit the role of accessory proteins of the gH/gL complex in the presence of an intact RL13 gene. Remarkably, in a recent *in vivo* study the UL74 homologue of the murine cytomegalovirus, m74, was required only for the initial cell-free infection of organs but was dispensable for the subsequent focal spread within the tissues of infected organs (Lemmermann *et al.*, 2015). This finding further emphasizes the importance of analysing the role of these complexes under conditions of clinical isolate-like cell-associated growth.

## Results

### Generation of the wild-type virus Merlin-RL13_tetO_ and introduction of stop mutations into UL74

Work with clinical HCMV isolates is challenging as their typical cell-associated phenotype is lost within a few passages in cell culture (Sinzger *et al.*, 1999). This is due to mutations in the RL13 gene and the UL128 gene locus, which allow for cell-free virus transmission and, hence, provide a selective advantage (Dargan *et al.*, 2010). Attempts to reconstitute a genetically complete version of the Merlin strain from a BAC clone regularly resulted in the *de-novo *selection of mutations in both RL13 and the UL128 locus (UL128, UL130 and UL131A). Protection of RL13 and the UL128 locus against this counterselection could be achieved by introducing tet operators upstream of each gene locus, and reconstituting virus in cells that constitutively express the tet repressor, thereby reducing expression of both gene loci. In this manner, genetically intact viruses can be generated (Stanton *et al.*, 2010). The addition of doxycycline results in sequestration of the repressor, and gene expression is restored. The virus would then behave like a clinical isolate, growing in a cell-associated manner for a limited number of passages.

**Fig. 5.**
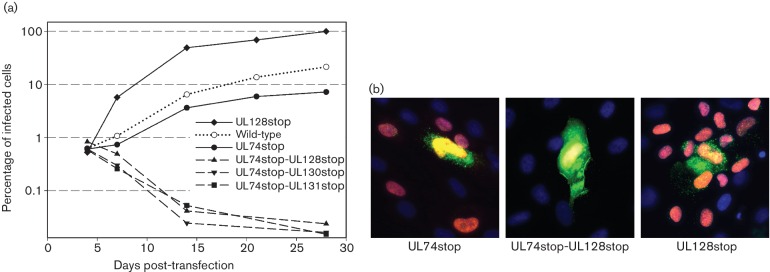
Phenotype of dual disruption of UL74 and the UL128 locus. (a) Human foreskin fibroblasts (HFFs) were transfected with equal amounts of DNA from wild-type, single or dual stop mutants. At indicated time points post-transfection, aliquots of the cultures were stained for viral immediate early (IE) antigens. The percentage of IE antigen-positive cells is plotted against time post-transfection. Only wild-type viruses and single mutants could grow and increase the number of IE antigen-positive cells over time, whereas none of the dual mutants could grow despite initial transfection rates being comparable to that of the single mutants. Data represent mean values of at least three repeated experiments for wild-type viruses, UL74stop mutant and UL128stop mutant. Data of the dual stop mutants are mean values from two independent clones for each mutant. (b) Immunofluorescence stainings of HFF cultures 7 days after transfection with the respective Merlin-BAC. Cells were fixed with acetone and stained by indirect immunofluorescence for IE antigens (red) and the late tegument protein pp150 (green).

In the background of this genetically complete Merlin-RL13_tetO_-UL128_tetO_ virus, we wanted to analyse the role of the UL74 reading frame that encodes for gO. However, a UL74 deletion mutant would be unlikely to be reconstituted in HFFF-tet cells (where the UL128 locus is repressed), since dual deletion of trimer and pentamer was lethal in our previous experiments in the genetic background of strain TB40/E (Jiang *et al.*, 2008). (Formal proof that dual disruption of both UL74 and the UL128 locus is actually lethal also in the Merlin background is provided by the data shown in Fig. 5.) Therefore, we removed the tet operator upstream of the UL128 region via markerless mutagenesis (Tischer *et al.*, 2010), leaving only RL13 regulated by the tet repressor. When the resulting BAC Merlin-RL13_tetO_ was transfected into HFFF-tet cells it formed foci of infected cells and could be grown to 10–20 % cytopathic effect (CPE) within five passages. To minimize the risk of mutations in RL13 or the UL128 locus, reconstituted viruses were only used for focus expansion experiments until 19 days post-transfection. The integrity of these genes was checked in each experiment by sequencing DNA from infected cells, and no mutations were found.

**Fig. 1.**
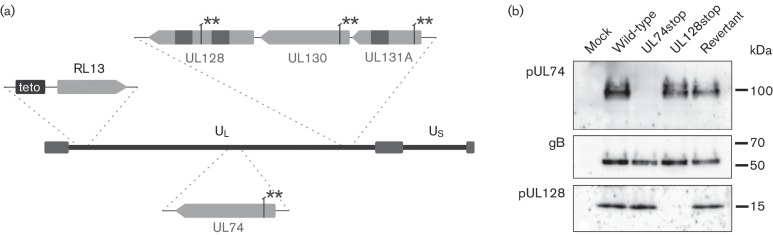
Characterization of mutants. (a) Overview of the relevant genomic modifications of the Merlin mutants generated in this study: all mutants carry the tet operator upstream of RL13. Two consecutive stop codons (**) were introduced into ORFs UL74, UL128, UL130 and UL131A as indicated in the designation of the respective mutants. (b) Expression of pUL74 and pUL128 in Merlin mutants. HFFF-tet cells were infected with the respective virus or mock-infected and analysed by immunoblotting to test for pUL74 and pUL128 expression. gB signals were used as a loading control. Numbers on the right side indicate the molecular mass. UL, unique long; US, unique short.

In the backbone of our wild-type virus Merlin-RL13_tetO,_ we knocked out pUL74 by introducing two stop codons at amino acid positions 16 and 21 via markerless mutagenesis (Tischer *et al.*, 2010) resulting in BAC Merlin-RL13_tetO_-UL74stop (Fig. 1a). This approach was chosen to disrupt expression of the UL74 gene product gO while minimizing undesired effects on neighbouring genes. With regard to UL73, the mutations are not located within the UL73 ORF but in the intron of primary UL73 transcripts. In contrast to our previous ATG deletion mutations (Jiang *et al.*, 2008), the UL74stop mutations would not affect the splice acceptor site of these pUL73-encoding transcripts (Gatherer *et al.*, 2011; Scalzo *et al.*, 2009). A BAC, in which these stop mutations were reverted, served as a control for effects caused by unwanted second site mutations. Both viruses could be reconstituted by transfection of the respective BAC-DNA into HFFF-tet cells and propagated by the passaging of the transfected cell cultures. Aliquots of the infected cultures were then stored and used for focus expansion assays. Disruption of protein expression was confirmed by Western blot analysis (Fig. 1b).

### Minor contribution of pUL74 to focal spread of the genetically complete HCMV strain Merlin

**Fig. 2.**
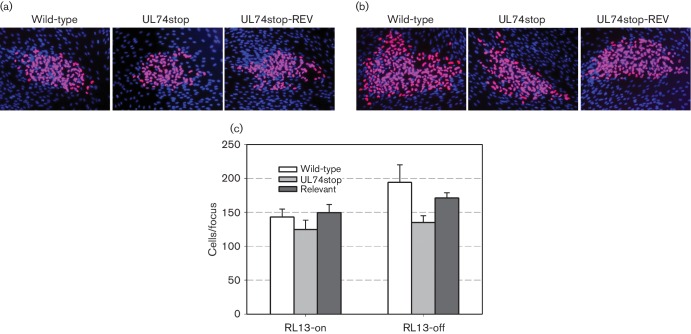
Effect of UL74 disruption on focal spread. Merlin-RL13_tetO_ (wild-type), Merlin-RL13_tetO_-UL74stop (UL74stop) and Merlin-RL13_tetO_-UL74stop-REV (Revertant) were reconstituted by transfection of the respective BAC-DNA into HFFF-tet cells. Infected HFFF-tet cells were then co-cultured with an excess of uninfected HFFF-tet cells for 7 days with or without doxycycline in the presence of human HCMV-negative serum. Cells were then fixed with 80 % acetone, viral IE-antigens were visualized by indirect immunofluorescence, and the mean number of infected cells per focus was determined. (a, b) Representative micrographs of foci (a) in the presence of doxycycline, i.e. under conditions of RL13 expression or (b) in the absence of doxycycline, i.e. under conditions of RL13 repression. (c) Graphical representation of focus size (mean±sem) as determined in 4 (RL13-on) or 3 (RL13-off) independent experiments.

Freshly reconstituted viruses Merlin-RL13_tetO_ (wild-type), Merlin-RL13_tetO_-UL74stop (UL74stop) and Merlin-RL13_tetO_-UL74stop-REV (revertant) were analysed by a co-culture-based focus expansion assay. Briefly, infected HFFF-tet cells were seeded together with a 100-fold excess of non-infected indicator cells and co-cultured for 7 days in the presence of doxycycline. Under these conditions RL13 is expressed, and as expected no infectious progeny was detected in the supernatant of these cultures, confirming the cell-associated phenotype of the RL13-expressing Merlin (Table 1). After 7 days, cultures were fixed, infected cells were stained by indirect immunofluorescence against viral IE antigens and the mean number of infected cells/focus was determined for each virus (Sinzger *et al.*, 1997). The plaque size of UL74stop (125 cells/focus) appeared reduced by 15 % when compared with wild-type (143 cells/focus) or revertant (150 cells/focus) but this reduction did not reach significance in four independent assays when compared with the mean values of wild-type and revertant (*P*=0.069) (Fig. 2a, c).

**Table 1.** Detection of cell-free infectious virus (infectious units ml^−1^) in the supernatant of infected cultures

Virus	Without DOX (RL13 repressed)	With DOX (RL13 expressed)
Wild-type	No virus	No virus
UL74stop	No virus	No virus
UL128stop	5.6×10^4^	4.6×10^2^

**Table 2.** Mutants used in this study

Mutant BAC	Genotype	Primer sequences*
Merlin-RL13_tetO_	Wild-type	GCCGCATGTTGCAGACTGAGAAAGAAAGCTTTATTATGAGACATCATACACATAGTATAGaggatgacgacgataagt TCCCCGCCCCATCACCTCGCCTATACTATGTGTATGATGTCTCATAATAAAGCTTTCTTTcaaccaattaaccaattctga
Merlin-RL13_tetO_-UL74stop	UL74stop	AAAAAAGAGATGATAATGGTGAAAGGCATTCCTAAAATT**TAA**CTCCTGATCTCT**TGA**ACGTTCTTGCTCCTTTCCCTaggatgacgacgataagt CCAATACATTACAATTTATGAGGGAAAGGAGCAAGAACGT**TCA**AGAGATCAGGAG**TTA**AATTTTAGGAATGCCTTTCAcaaccaattaaccaattctga
Merlin-RL13_tetO_-UL128stop	UL128stop	ACGGCTGAGATTCGCGGGATCGTCACCACCATGACC**TAG**TCATTGACA**TGA**CAGGTCGTACACAACAaggatgacgacgataagt GTAGTTGCAGCTCGTCAGTTTGTTGTGTACGACCTG**TCA**TGTCAATGA**CTA**GGTCATGGTGGTGACGcaaccaattaaccaattctga
Merlin-RL13_tetO_-UL74stop-UL128stop	UL74stop-UL128stop	ACGGCTGAGATTCGCGGGATCGTCACCACCATGACC**TAG**TCATTGACA**TGA**CAGGTCGTACACAACAaggatgacgacgataagt GTAGTTGCAGCTCGTCAGTTTGTTGTGTACGACCTG**TCA**TGTCAATGA**CTA**GGTCATGGTGGTGACGcaaccaattaaccaattctga
Merlin-RL13_tetO_-UL74stop-UL130stop	UL74stop-UL130stop	CTGCCTGCTTCTGTGCGCGGTTTGGGCAACGCCCTGTCTG**TAG**TCTCCGTGGTCG**TAA**CTAACAGCAAACCAGAATCCaggatgacgacgataagt GTTTAGACCATGGCGGGGACGGATTCTGGTTTGCTGTTAG**TTA**CGACCACGGAGA**CTA**CAGACAGGGCGTTGCCCAAAcaaccaattaaccaattctgaPlos
Merlin-RL13_tetO_-UL74stop-UL131stop	UL74stop-UL131stop	GTCTGTTTGTCTGTGCGCCGTGGTGCTGGGTCAGTGCCAG**TAG**GAAACCGCGGAA**TAA**AACGATTATTACCGAGTACCaggatgacgacgataagt AGCACGCGTCCCAGTAATGCGGTACTCGGTAATAATCGTT**TTA**TTCCGCGGTTTC**CTA**CTGGCACTGACCCAGCACCAcaaccaattaaccaattctga
Merlin-RL13_tetO_-UL74stopREV-UL128stopREV	Revertant	AAAAAAGAGATGATAATGGTGAAAGGCATTCCTAAAATT**ATG**CTCCTGATCTCT**ATA**ACGTTCTTGCTCCTTTCCCTaggatgacgacgataagt CCAATACATTACAATTTATGAGGGAAAGGAGCAAGAACGT**TAT**AGAGATCAGGAG**CAT**AATTTTAGGAATGCCTTTCAcaaccaattaaccaattctga ACGGCTGAGATTCGCGGGATCGTCACCACCATGACC**CAT**TCATTGACA**CGC**CAGGTCGTACACAACAaggatgacgacgataagt GTAGTTGCAGCTCGTCAGTTTGTTGTGTACGACCTG**GCG**TGTCAATGA**ATG**GGTCATGGTGGTGACGcaaccaattaaccaattctga

*Uppercase letters, HCMV homology; lowercase letters, homology to template plasmid pEP-Kan-S; bold letters, sequence of interest.

To analyse the effects of UL74 in the absence of RL13, replica cultures were included in each experiment that were identical except for the omission of doxycycline. In the absence of doxycycline RL13 is repressed, and this might allow for release of infectious progeny. If so, based on previous data with other HCMV strains (Jiang *et al.*, 2008; Wille *et al.*, 2010), deletion of UL74 should have a significant effect on spreading efficiency. However, there was still no infectious virus detectable in the supernatant, indicating that repression of RL13 was not sufficient to overcome the cell association of Merlin-RL13_tetO_ in our culture system. When UL74 was deleted in the absence of RL13, there was a 26 % reduction of plaque size (135 cells/focus) when compared with wild-type (195 cells/focus) or revertant (170 cells/focus), and this difference just reached significance in three independent experiments (*P*=0.049) (Fig. 2b, c).

To evaluate the effect of RL13 on focal spread, all three experiments without doxycycline (RL13-off) and the respective three matched experiments with doxycycline (RL13-on) were statistically analysed by a *t*-test for paired samples. In this reduced set of RL13-on experiments the mean focus size of wild-type, UL74stop and revertant was 134, 112 and 143 cells/focus, respectively. In comparison, under RL13-off conditions the mean focus size (195, 135 and 170 cells/focus, respectively) was increased for each virus by 44 %, 20 % and 21 %, respectively. Taken together, the effect of RL13 was highly significant (*P*=0.003), confirming previous reports of the restrictive effect of this gene on HCMV replication.

To rule out an effect of doxycycline itself on focus size, we compared focal growth of wild-type virus in primary fibroblasts [human foreskin fibroblasts (HFFs)] in the absence or presence of doxycycline. As these cells do not express the tet repressor, RL13 is expressed irrespective of doxycycline; and any effect of doxycycline in this setting would indicate a proprietary effect of this drug on focus size. However, neither doxycycline itself nor its solvent ethanol had an effect on focus size in this setting (Fig. S1, available in the online Supplementary Material).

**Fig. 3.**
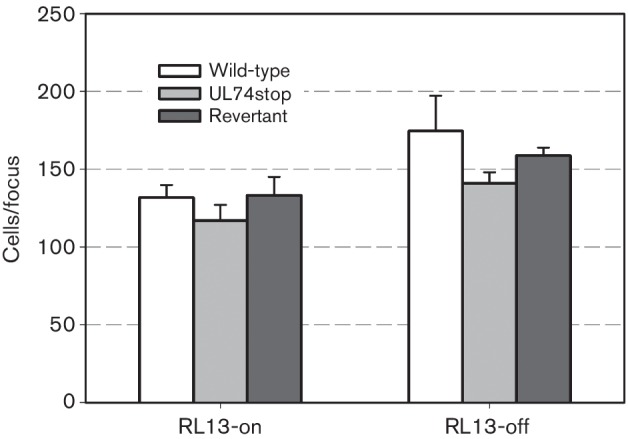
Effect of UL74 disruption on focal spread in the presence of human anti-HCMV serum. Infected HFFF-tet cells were co-cultured with an excess of uninfected HFFF-tet cells for 7 days with or without doxycycline in the presence of human HCMV-positive serum at a concentration sufficient to neutralize cell-free virions. Cells were then fixed with 80 % acetone, viral IE-antigens were visualized by indirect immunofluorescence and the mean number of infected cells per focus was determined. Graphical representation of focus size (mean±sem) as determined in four (with DOX) or three (without DOX) independent experiments.

The fact that detectable levels of cell-free virus were not found in the supernatant of the respective cultures argued against a major contribution of cell-free virus. This issue was further addressed by performing parallel experiments in the presence of neutralizing anti-HCMV serum to estimate the contribution of cell-free virus transmission. Co-culture of infected and uninfected cells was performed as before, except that serum was added at a concentration that was sufficient to completely neutralize a cell-free preparation of strain Merlin (Merlin-RL13_tetO_-UL128_tetO_ produced on HFFF-tet cells; Fig. S2). The overall plaque size of wild-type and revertant virus was reduced by <10 % in the presence of HCMV-seropositive serum (Fig. 3) when compared with results in the presence of HCMV-seronegative serum (Fig. 2c), which reached significance only when RL13 was repressed (*P*=0.033). Focal growth of UL74stop virus was not inhibited by HCMV-seropositive serum (*P*=0.959). Again, comparison of the three matched experiments showed an increased plaque size upon repression of RL13 for each of the viruses (by 37 %, 25 % and 32 %, respectively; *P*=0.001), providing evidence that RL13 restricts cell-associated growth of strain Merlin in the presence of HCMV seropositive serum also. While the serum-sensitive component of focal growth is somewhat suggestive of cell-free virus transmission even in the absence of detectable levels of cell-free infectivity, an alternative effect of the serum on cell-associated spread cannot be excluded.

**Fig. 4.**
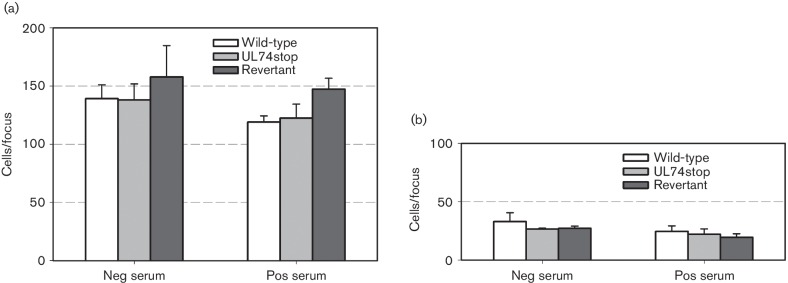
Effect of UL74 disruption on focal spread in primary cells. Merlin-RL13_tetO_ (wild-type), Merlin-RL13_tetO_-UL74stop (UL74stop) and Merlin-RL13_tetO_-UL74stop-REV (Revertant) were reconstituted by transfection of the respective BAC-DNA into HFF cells. Infected HFF cells were co-cultured with an excess of uninfected HFFs (a) or HUVECs (b) for 7 d in the presence of human HCMV-negative or -positive serum. Cells were then fixed with 80 % acetone, viral IE-antigens were visualized by indirect immunofluorescence, and the mean number of infected cells per focus was determined. Graphical representation of focus size (mean±sem) as determined in three independent experiments.

To exclude the possibility that a more prominent effect of UL74 is masked in the immortalized HFFF-tet cells, we repeated the same experiments in primary fibroblasts and endothelial cells [human umbilical vein endothelial cells (HUVECs)]. These cells do not express the tet repressor, and RL13 expression is hence unrestricted. As in HFFF-tet cells, HCMV-seropositive serum had a moderate inhibitory effect, which was more pronounced in HUVECs (27 %) as compared with HFFs (10 %), but was not significant in the three independent experiments in either cell type (*P*=0.100 and 0.356, respectively) (Fig. 4). Irrespective of whether HCMV-seropositive or HCMV-seronegative serum was added, disruption of UL74 had no significant effect on plaque size in either cell type.

In conclusion, UL74 has only a marginal, if any, effect on cell-associated growth of a genetically complete HCMV strain, and focal growth of this virus is mostly resistant to anti-HCMV serum, particularly in fibroblasts.

### Disruption of the UL128 locus reveals the contribution of pUL74 to cell-free transmission

The lack of a significant contribution of UL74 to growth of the genetically complete Merlin in fibroblasts and endothelial cells raised doubts as to whether gO expressed by this strain was functional at all. We hypothesized that a hidden function of the trimeric gH/gL/gO complex would be revealed when disruption of the UL128 locus abolished expression of the pentameric gH/gL/pUL128-131A complex.

Previous work in the background of the cell-culture adapted strain TB40/E had shown that the combined disruption of UL74 and the UL128 locus is lethal, indicating that accessory proteins are essential for the function of gH/gL in the context of HCMV (Jiang *et al.*, 2008). However, since a single deletion of UL74 did not substantially alter the spread of our genetically intact Merlin-BAC derived virus and a single deletion of UL128 actually enhances virus spread (Stanton *et al.*, 2010), it was possible that the effect of simultaneous deletion of both gene regions would differ in this virus. For example, gH and gL alone might form functional heterodimers, or even homodimers of two gH/gL molecules (Fouts *et al.*, 2014). We therefore knocked out both UL74 and the UL128 locus in the background of Merlin-RL13_tetO_.

In the background of Merlin-RL13_tetO_-UL74stop, two stop codons were introduced into each of the genes within the UL128 locus, resulting in mutants Merlin-RL13_tetO_-UL74stop-UL128stop, Merlin-RL13_tetO_-UL74stop-UL130stop and Merlin-RL13_tetO_-UL74stop-UL131stop. In addition, a Merlin-RL13_tetO_-UL128stop mutant was generated to serve as a control in which only the pentamer is disrupted but the trimer is intact. Equal amounts of BAC-DNA of the various mutants were then transfected into HFF cultures, and the reconstitution of the respective viruses was monitored by quantitative detection of IE-antigen-positive cells in the transfected cultures for an extended period of 28 days, to detect growth of the dual mutant in case it occurred with a delay. The initial transfection rate was similar with all mutants, with about 1 % of cells expressing IE antigen. While the number of infected cells increased over time with viruses expressing either the pentamer or the trimer or both, indicating successful replication, the number of infected cells decreased with all dual mutants, suggestive of abortive infection (Fig. 5a). Ultimately, both Merlin-RL13_tetO_-UL74stop and Merlin-RL13_tetO_-UL128stop formed foci of infected cells, whereas the dual mutants yielded only single-standing infected cells that expressed IE antigens and late antigens but could not spread the infection, indicated by lack of IE antigens in neighbouring cells (Fig. 5b). The revertant virus generated on the basis of Merlin-RL13_tetO_-UL74stop-UL128stop grew like the parental Merlin-RL13_tetO_, ruling out the possibility that the non-viable phenotype of the dual mutants was due to unwanted second site mutations (data not shown). Thus, as in TB40/E, dual disruption of UL74 and the UL128 locus is lethal in Merlin.

The UL74stop mutant showed a small reduction regarding viral growth during the reconstitution period when compared with wild-type virus (Fig. 5a), which was consistent with the minor contribution of UL74 to focal growth seen in the previous section. Remarkably, the UL128stop mutant grew faster than wild-type virus, and this superiority of the UL128stop mutant over wild-type virus was addressed in a final set of experiments.

**Fig. 6.**
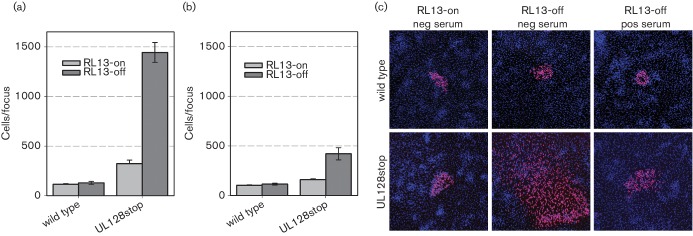
Effect of UL128 disruption on focal spread. Infected HFFF-tet cells were co-cultured with an excess of uninfected HFFF-tet cells for 7 days with (RL13-on) or without (RL13-off) doxycycline in the presence of human HCMV-negative (a) or HCMV-positive (b) serum. Cells were then fixed with 80 % acetone, viral IE-antigens were visualized by indirect immunofluorescence, and the mean number of infected cells per focus was determined. Graphs represent the focus size (mean±sem) as determined in three independent experiments. (c) Representative micrographs showing focal growth of wild-type virus (Merlin-RL13_tetO_) and the UL128stop mutant under various conditions.

The fact that Merlin-RL13_tetO_-UL74stop-UL128stop could not be reconstituted indicated that the viral spread depended totally on the expression of either the pentamer or trimer. Hence, the extent and mode of virus transmission of the UL128stop mutant can be unequivocally attributed to expression of UL74. Previous reports suggested that UL74 is critical for cell-free virus transmission. To test whether UL74 also mediates cell-free transmission in the background of strain Merlin, the UL128stop virus was compared to wild-type virus with and without RL13 expression regarding focus size in the absence or presence of anti-HCMV serum. Serum-resistant growth is assumed to reflect cell-associated spread, whereas serum sensitive growth is assumed to reflect cell-free transmission. The UL128stop mutant grew with increased plaque size as compared with wild-type, and this gain of spreading capacity was greatly enhanced when RL13 was downregulated (Fig. 6a). The spread of the mutant was predominantly serum-sensitive, indicating cell-free transmission via the trimer (Fig. 6b vs Fig. 6a); however, a serum-resistant residual focus remained, indicative of cell-associated spread. This result was corroborated in an additional experiment comparing the effects of HCMV-positive serum and a neutralizing monoclonal anti-gB antibody on focal spread of the UL128stop mutant, both showing the same degree of inhibition (Fig. S3). The appearance of a comet shape under conditions of RL13 downregulation supported the idea that a large part of the trimer-mediated growth was due to cell-free virus transmission (Fig. 6c). Consistent with this assumption, this comet-shaped spread was mostly neutralized by anti-HCMV serum. Finally, the question of whether Merlin-RL13_tetO_-UL128stop releases infectious progeny was directly addressed by the analysis of supernatants from infected cultures. Cell-free infectivity was found with Merlin-RL13_tetO_-UL128stop, with the amount of infectivity depending on RL13 repression (Table 1). Thus when RL13 was expressed (in the presence of doxycycline), titres were 100-fold lower as compared with titres in the presence of RL13 (i.e. in the absence of doxycycline). Taken together, these findings indicate that the trimer can drive both cell-associated (serum-resistant) and cell-free transmission (serum-sensitive) with RL13 being an effective restriction factor of cell-free UL74-driven spread (also see Fig. 6c).

## Discussion

One major conclusion from our results is that disruption of UL74 has little, if any, effect on focal cell-associated spread of the genetically complete variant of HCMV strain Merlin, in either fibroblasts or endothelial cells. There was a slight reduction by some 15 %; however, this was not highly significant in a set of four experiments. This contrasts with the well-documented small plaque phenotype that is caused by disruption of UL74 in the background of various cell culture-adapted HCMV strains (i.e. strains that can form large comet-shaped foci via cell-free virus) including AD169, Towne, TR and TB40-BAC4 (Dunn *et al.*, 2003; Hobom *et al.*, 2000; Jiang *et al.*, 2008; Wille *et al.*, 2010; Yu *et al.*, 2003). In these strains, the small plaque phenotype can be explained by an almost complete loss of cell-free spread of viral progeny (Jiang *et al.*, 2008; Wille *et al.*, 2010). Thus it may be that loss of pUL74 failed to have a similar effect on focus size in the context of Merlin since (like clinical isolates) Merlin does not produce large amounts of cell-free virus in culture (Dargan *et al.*, 2010; Stanton *et al.*, 2010).

We can exclude the possibility that the UL74 gene product is non-functional in our wild-type virus Merlin-RL13_tetO_ as its contribution was revealed in the absence of expression from the UL128 locus. Thus it appears that both UL74 and the UL128 locus can mediate viral spread, though via different mechanisms. The spread driven by the UL128 locus, probably via the pentameric gH/gL complex, appears cell-associated and almost completely resistant to neutralizing anti-HCMV serum, whereas the spread driven only by the trimer has a cell-free component as indicated by its susceptibility to neutralizing anti-HCMV serum and the detection of infectious progeny in the supernatant.

Regarding the specific contributions of the trimer and pentamer in the virion envelope, it seems that levels of one complex are always formed at the expense of the other, since both gO and UL128 bind to the same cysteine on gL (Ciferri *et al.*, 2015; Zhou *et al.*, 2015). Thus in the genetically intact Merlin, levels of pentamer are high, but trimer is low, and loss of gO has little effect. Under these circumstances, the spread is pentamer-driven and is almost exclusively cell-associated. In strains that have been passaged, genetic changes result in increased levels of the trimer, but reduced levels of pentamer (Murrell *et al.*, 2013; Zhou *et al.*, 2013, 2015). When this occurs, cell-free infectivity appears, and this transmission mode depends on UL74. Consistent with our previous findings in the TB40/E background (Jiang *et al.*, 2008), gH and gL alone were not sufficient for virus spread in the context of Merlin, arguing against a role for gH-gL dimers in the spread, at least in the cell types tested here.

While pentamer-mediated spread is clearly almost exclusively cell-associated, the situation is not as unequivocal with the trimer. There is certainly a strong cell-free component as proven by the detection of infectivity in the supernatant of mutant Merlin-RL13_tetO_-UL128stop and the inhibitory effect of anti-HCMV serum on the plaque size of this virus. However, there is also substantial spread in the presence of the neutralizing serum indicating that there is also a cell-associated component. Fitting with this interpretation, the serum-resistant component of plaques mediated by the trimer have a similar size as the pentamer-driven cell-associated plaques.

While this study was primarily focussed on the role of gO in the context of a genetically complete HCMV strain, it also confirms the restrictive effects of RL13 on viral spread. The plaque size of all tested viruses was increased when RL13 was repressed, fitting in with the reported function of RL13 as an inhibitor of virus spread (Stanton *et al.*, 2010). When the pentamer was disrupted by stop mutations in the UL128 locus, cell-free virus transmission occurred, and in this situation RL13 greatly restricted the amount of cell-free infectivity, fitting in with previous reports on the effects of RL13 mutations on virus titres (Dargan *et al.*, 2010; Stanton *et al.*, 2010). It is remarkable that HCMV encodes two factors that can restrict cell-free virus transmission, RL13 and the UL128 region. As a consequence, effective cell-free spread only occurs when both gene regions are downmodulated.

It is an unusual situation that of the three gene regions addressed in this study, RL13 and UL128 restrict viral replication and UL74 is dispensable when the other two are expressed, but none clearly increases replication in the context of a genetically complete HCMV. This raises several questions: Why are these genes conserved among HCMV isolates, i.e. what is their function during the natural course of infection in the host? Are there certain cell types or tissues in which they promote viral replication? Is their replication-promoting effect only revealed in the presence of an antiviral immune response? The mutants created on the basis of the genetically complete Merlin-BAC can now be applied to address these questions.

Considering our data in the context of existing literature on gO, it is tempting to speculate about the possible role of the trimeric gH/gL complex during natural infection. Homologues of gO are conserved among beta herpesviruses, indicating that it performs a crucial function. Considering only HCMV strains, there is a remarkable degree of polymorphism, suggesting a selective pressure on this protein (Mattick *et al.*, 2004; Paterson *et al.*, 2002; Rasmussen *et al.*, 2002; Stanton *et al.*, 2005). Taken together, it appears unlikely that gO is dispensable *in vivo*. Rather, the retention of UL74 by the virus suggests that cell-free virus is important for the biology of the virus. A hint at a possible function comes from recent work with murine cytomegalovirus (MCMV), where gH/gL also forms a trimer with gO and an alternative complex with MCK-2 (encoded by m131–129). In an* in vivo* model both the efficiency of initial inoculation of mice with cell-free virus (intraperitoneally or intravenously) and subsequent spread within various organs were evaluated (Lemmermann, *et al.*, 2015). In this model, gO was necessary for cell-free infection of a new host, but dispensable for spread within organ tissues. It is tempting to assume a similar function of gO for HCMV: the trimeric complex would then be essential for cell-free host-to-host spread, e.g. by breast milk, saliva or urine, whereas cell-associated dissemination within the infected host would be primarily driven by the pentameric complex. In this context it is noteworthy that cell-free infectious HCMV can be found in the milk whey of lactating women and in the urine of infected children (Hamprecht *et al.*, 2003; Stagno *et al.*, 1975), but once they are isolated and propagated in fibroblast cultures HCMV strains are cell-associated (Sinzger *et al.*, 1999; Yamane *et al.*, 1983). Speculating about possible sources for the cell-free infectivity in urine and milk whey, epithelial cells in the kidney and the breast are among the primary candidates. Interestingly, cell type differences regarding virus release and virion composition have been reported previously (Scrivano *et al.*, 2011; Wang *et al.*, 2007), suggesting that the search for a cell type that would transmit the genetically complete Merlin in a cell-free mode, dependent on gO, is a realistic target.

## Methods

### Cells and viruses.

HFFF-tet cells (Stanton *et al.*, 2010) and primary human foreskin fibroblasts (HFF) were cultured in minimum essential medium (MEM) supplemented with GlutaMAX (Life Technologies), 5 % FCS, 0.5 ng ml^−1^ basic fibroblast growth factor (bFGF, Life Technologies), and 100 µg ml^−1^ gentamicin. Experiments were carried out in the absence of bFGF to exclude the possibility of interference of this growth factor with virus transmission. Human umbilical vein endothelial cells (HUVECs) were always seeded onto gelatin-coated vessels and maintained in RPMI1640 medium (Life Technologies) supplemented with 10 % HCMV-seronegative human serum, 50 µg ml^−1^ endothelial cell growth supplement (ECGS, BD Biosciences), 5 units ml^−1^ heparin (Sigma-Aldrich) and 100 µg ml^−1^ gentamicin.

A Merlin-BAC clone containing the complete wild-type HCMV strain Merlin genome, with tet-operators inserted upstream of UL131A and RL13 (Merlin-RL13_tetO_-UL128_tetO;_ pAL1502) (Stanton *et al.*, 2010) was used as the parental BAC in this study. Merlin-BAC and derived mutants were reconstituted in HFF or HFFF-tet cells by calcium phosphate transfection (MBS transfection kit, Agilent) or lipofection (K2 Transfection System, Biontex) and further propagated in the absence of bFGF. Infected cells were cultured up to 19 days post-transfection and frozen into aliquots. In parallel, a small portion of the culture was adhered to gelatin-coated 96-well plates and subjected to the determination of the infection rate by immediate early (IE) antigen staining.

### Generation of mutants.

Mutant BACs were generated by applying the markerless mutagenesis protocol of Tischer *et al.* (2010). In brief, Merlin-BAC-DNA was retransformed into the *Escherichia coli* strain GS1783 by electroporation, and the integrity of the genome was confirmed by restriction fragment length analysis (RFLA). To generate mutants, recombination fragments were generated by PCR from plasmid pEP-Kan-S with primers as shown in Table 2. The resulting fragments consisted of the 18-bp I-Sce I restriction site and a kanamycin resistance cassette flanked by repeated sequences containing homology to the desired site of insertion in the HCMV genome. The recombination fragments were inserted into the recombination-activated GS1783 harbouring the Merlin-BAC by electroporation. Following kanamycin selection, all non-HCMV sequences were removed by an intrabacterial I-Sce I digest and a subsequent red recombination step. BAC-DNA was isolated using the NucleoBond Xtra Midi kit (Macherey-Nagel), and each mutant was analysed by RFLA and sequencing.

### Immunofluorescence.

 For detection of viral IE antigen (pUL122/123), cells were fixed with 80 % acetone and incubated sequentially with primary antibody E13 (Argene) and secondary antibody Cy3-goat anti-mouse IgG F(ab′)_2_ (Jackson ImmunoResearch). Additional detection of viral pUL32 was achieved by incubation with primary antibody MAb 36–14 (a generous gift from W.J. Britt, University of Alabama, Birmingham) (Sanchez *et al.*, 2000) and secondary antibody Alexa Fluor488-conjugated goat anti-mouse IgG F(ab′)_2_ (Invitrogen). Counterstaining of nuclei was performed with DAPI.

### Focus expansion assays.

 Viral spread was tested by a focus expansion assay essentially as previously described (Sinzger *et al.*, 1997). Aliquots of infected cell cultures (HFFs or HFFF-tet cells with about 10 % CPE) were thawed, washed once with MEM and co-cultured with a 100-fold excess of uninfected indicator cells (HFFs, HFFF-tet cells or HUVECs) for 7 days in gelatin-coated 96-well plates in the presence of 2 % native human HCMV-negative or positive serum. In the case of HFFF-tet cells, infected cells and indicator cells were pretreated with 2 µg ml^−1^ doxycycline or its solvent ethanol for a minimum of 3 days prior to co-culture and maintained under treatment until termination of the experiment. At day three post-seeding, half of the supernatant was exchanged with freshly prepared medium containing doxycycline or ethanol together with the respective human seronegative or seropositive serum. Plates were fixed and stained for IE antigen and DAPI as described above. The average number of infected cells per focus was quantified. All experiments were controlled for the absence of unwanted mutations in relevant genes (RL13, UL74, UL128, UL130, UL131A) by PCR amplification followed by sequencing of DNA either from frozen aliquots used for co-culture or from stained cell cultures at the end of focus expansion assays.

### Detection of cell-free infectivity.

Supernatants from focal expansion assays treated with human HCMV-negative serum were harvested prior to termination of the experiment from four individual wells, pooled and centrifuged at 3220 ***g*** for 10 min to remove cellular debris. HFF indicator cells in gelatin-coated 96-well plates were infected with serial dilutions of the respective freshly prepared supernatants in duplicate and incubated for 24 h. Cells were fixed, stained for viral IE antigen as described above and viral titres were calculated as infectious units ml^−1^.

### Western blot analysis.

Aliquots of infected HFFF-tet cells were thawed, washed once with MEM and co-cultured with uninfected HFFF-tet cells in six-well plates for 7 days. Cells were scraped from the plates, pelleted and washed twice with PBS. Cell lysis was performed on ice in a buffer containing 2 % sodium dodecylsulfate, 62.5 mM Tris (pH 6.8), 10 % glycine and 0.01 % bromphenol blue. After addition of 10 % 2-mercaptoethanol, proteins were separated in 10 % polyacrylamide gels and transferred to polyvinylidene fluoride membranes (Millipore) in Tris-Glycine buffer (containing 38 mM Tris, 288 mM glycine and 15 % methanol). Membranes were blocked with PBS plus 0.1 % Tween and 5 % milk powder. Gel loads were adjusted to gB signals (mouse monoclonal anti-cytomegalovirus glycoprotein B antibody, abcam). The mouse monoclonal anti-pUL128 was a generous gift from Giuseppe Gerna (Pavia, Italy) (Gerna *et al.*, 2008). Anti-gO mouse mAb was generated by immunizing BALB/c mice with gO/human IgG1Fc fusion protein purified by protein A affinity chromatography from supernatants of transfected HEK293T cells as described previously (Jager *et al.*, 2013). Stable hybridoma cell lines were generated by fusing SP2/0 myeloma cells with spleen cells of an immunized mouse. The antibody was purified by protein G affinity chromatography using an ÄKTAprime plus system (GE Healthcare). HRP conjugated secondary antibodies were purchased from Santa Cruz. For detection, membranes were incubated with luminol (Super Signal West Dura chemiluminescence kit, Pierce) and signals were visualized in a chemiluminescence reader (Fusion SL, Peqlab).

### Statistical analyses.

Differences between paired data sets from 3–4 independent experiments were analysed for statistical significance with two-tailed *t*-tests for paired samples using the built-in data analyses function of Excel. Data shown in Figs 2(c) and 3 originate from the same set of experiments and could, hence, also be analysed in a pairwise fashion.
